# Satisfy Your Appetite

**Published:** 1889-10

**Authors:** 


### SATISFY YOUR APPETITE.

A physician writing on the food necessary to give strength and sustenance says that if a person uses up his brain faster than he makes it he soon becomes nervous and irritable. If he does not assimilate enough food to supply its demands his mind is sure to become weak. The healthiest and strongest individuals even should eat a far greater proportion of meat than of vegetable food. Beef should be taken as the standard meat. It answers every purpose of the system. Veal and pork are not as easily digested. Pork, so far as its composition goes, is an excellent food for nervous persons, but it is not readily digested. Yet, in the army, we used to think nothing better for the wounded men than bacon. As a rule salt meat is not adapted to the requirements of the nervous individual, as nutritious juices to a great extent go into the brine.

The flesh of wild birds is more tender and more readily digested than that of domestic ones. This is accounted for by the greater amount of exercise they take, thereby renewing their flesh more rapidly and making it younger than that of birds which lead a more quiet life. This is a suggestiou that might be of benefit to women of sedentary habits who are desirous of prolonging an appearance of youth. Fish of all kinds is a good food for the nervously inclined. Raw eggs, contrary to general opinion, are not as digestible as those that have been cooked.

A notion has been prevalent that many persons injure their digestion by eating too much. The fact is that most people don't eat enough. There are more people killed every year by insufficiency of nourishment than by overloading their stomachs. Many of those who do eat a sufficient quantity are prevented by disease from digesting enough for the economy of their systems. The very first thing for any one to do who has exhausted himself by mental work or who has been born weak and irritable is to furnish his brain with sufficient nourishment to either repair the damage it has sustained or to build it into a strong, healthy condition. People in this condition usually suffer from nervous dyspepsia. Their stomachs are unable to perform the labor of assimilation. Owing to the deficient nerve power of the individual the food lies in the stomach unacted upon by the gastric juice because there is none or the quantity is insufficient to have any power. The food;instead of helping to renew the body, and the nervous system with the rest, undergoes fermentation,and the body and brain it should nourish may starve,and the person is in a worse state than if the food had not been taken, for the fermentation generates acids and gas.

Nervous individuals may derive all the fat they need from sugar and starch. It is better, however, for those with weak digestive organs, or whose nerves are in a highly sensitive state, to get it from the animal kingdom than compel their enfeebled stomachs, intestines and pancreas to create it out of these articles. Good bread, sweet butter and meat are the best foods for the nerves.

People troubled with insomnia, nervous starting from sleep and sensations of falling, can often be cured by limiting themselves to a diet of milk alone for a time. An adult should take a pint for a meal and take four meals daily. People with weakened nerves require usually a larger quantity of water than those whose brains and nerves are strong. It aids in the digestion of food by making it soluble and seems to have a direct tonic effect.

With proper eating and drinking we should have fewer broken-down nervous wrecks, and far more vigorous intellects. The present human species cannot eliminate flesh from its food and amount to a row of pins. The fancy that nothing but vegetables should be eaten is apt to overtake every one somewhere in life. It is due to some disorganization and usually passes away with the disturbance that creates it.—*Chicago Globe.*

The question of beauty takes us out of surfaces, to thinking of the foundations of things. The tint of the flower proceeds from its root, and the lusters of the sea shell begin with its existence.—*Emerson.*

